# The antibody response of haematological malignancies to COVID-19 infection and vaccination

**DOI:** 10.1038/s41416-021-01682-6

**Published:** 2022-01-11

**Authors:** Nicole A. Seebacher

**Affiliations:** Department of Oncology, University of Oxford, Oxford, UK

**Keywords:** Haematological cancer, Cancer therapy

## Abstract

Cancer patients with COVID-19 have reduced survival. While most cancer patients, like the general population, have an almost 100% rate of seroconversion after COVID-19 infection or vaccination, patients with haematological malignancies have lower seroconversion rates and are far less likely to gain adequate protection. This raises the concern that patients with haematological malignancies, especially those receiving immunosuppressive therapies, may still develop the fatal disease when infected with COVID-19 after vaccination. There is an urgent need to develop Guidelines to help direct vaccination schedules and protective measures in oncology patients, differentiating those with haematological malignancies and those in an immunocompromised state.

As severe acute respiratory syndrome coronavirus 2 (SARS-CoV-2) continues to spread globally at an alarming rate, it is having an unprecedented impact on cancer patients [1]. Much of the focus in the available literature has highlighted the burden that the coronavirus disease 2019 (COVID-19) pandemic has placed on cancer care, including delaying diagnoses and treatment and halting clinical trials. As more data become available, we are seeing that oncology patients have worse outcomes to the COVID-19 infection, including greater incidence of acute respiratory distress syndrome and higher morbidity and mortality rates [2–4]. Studies have revealed reduced survival from COVID-19 in highly susceptible cancer patients, including those with advanced age, harbouring multiple comorbidities, or where the cancer is haematological [3, 5]. In a study of 3377 patients with haematological malignancies, the risk of death with COVID-19 was 34%, which is markedly higher than the 4.8% reported with solid tumours [6].

While most cancer patients, like the general population, have an almost 100% rate of seroconversion after COVID-19 infection or receiving mRNA or adenovirus-based COVID-19 vaccines, patients with haematological malignancies, most notably those receiving anti-CD20 immunotherapy, have lower rates of seroconversion and are far less likely to gain adequate protection [2, 7]. In a study of 200 patients with cancer, seroconversion rates of 94, 85 and 70% were reported in patients with solid cancers, haematological malignancies, and haematological malignancies receiving anti-CD20 therapies, respectively [2, 3]. Similar results have been seen in a retrospective study of 160 adults with haematological malignancies vaccinated in the U.S. in early 2021 [7]. Interestingly, a recent study reported that rituximab prevents the anti-SARS-CoV-2 humoral response for at least 6 months after recovery from COVID-19 infection [8]. This raises the concern that patients with haematological malignancies, especially those receiving immunosuppressive therapies, may still develop the fatal disease when infected with COVID-19 after vaccination. However, while this may be clinically significant, it is unclear how seroconversion correlates with clinical outcomes. Two *Nature Medicine* publications have recently reported that the COVID-19-vaccine efficacy can be linked to neutralising antibody titre [9].

There is now available data demonstrating an improved serological response to a third dose of the mRNA COVID-19 vaccine in select immunocompromised groups, including solid-organ transplant recipients, renal-dialysis patients, and patients with haematological malignancies, most notably those who had not received anti-CD20 therapy within a year [10]. Many countries now recommended an additional booster in select immunocompromised cohorts. However, it is important to note that those who did not have an antibody response after two vaccine doses remained seronegative after their third dose of the same vaccine [10].

While the timing of anti-CD20 therapy impacts the humoral response, it does not modify the T cell response [10]. Given the importance of a T cell response in COVID-19, the emergence of a specific T cell response is another expected benefit of a booster vaccine, especially for patients receiving anti-CD20 therapy [10]. Therefore, the data support using a booster vaccine in immunocompromised patients, accepting that some individuals will still have vaccine failure. Studies are underway to identify if using a different vaccine type as a booster, “heterologous boosting”, may help produce antibodies [11].

Understanding the impact of immunosuppression on the effectiveness of these vaccines highlights the need for other prophylactic strategies in this immunosuppressed population to mitigate COVID-19 infection or by boosting the immune system response with unique vaccine schedules [2]. Unfortunately, most COVID-19 clinical trial studies to date have excluded patients diagnosed with a malignancy. Therefore, there is minimal information on the safety and efficacy of these vaccines in this population [12].

Position statements and guidelines for COVID-19 vaccinations in individuals with cancer receiving anti-cancer therapies are frequently released. While many suggest that COVID-19 vaccinations be administered two weeks or more prior to chemotherapy, this recommendation has not been practical. Limited by vaccination availability and the difficulties surrounding the scheduling of chemotherapy around the need for two vaccinations [13]. The push for earlier vaccine administration in these groups has been the priority. Advice from the British Society for Haematology provides guidance for clinicians caring for patients with blood cancers, including the most up to date information from the National Cancer Research Institute.

Decisions surrounding the appropriateness of the COVID-19 vaccine for individuals affected by cancer are currently made on an individual basis by their healthcare team. Patients must be counselled about the unknown vaccine safety profile and effectiveness in immunocompromised populations, the potential for reduced immune responses and the need to continue to follow all current guidance to protect themselves against COVID-19. Therefore, there is a need to develop Policies and Guidelines to help direct vaccination administration schedules in oncology patients, differentiating those with haematological malignancies and those in an immunocompromised state. Interestingly, vaccine-safety information is also absent in the context of therapies that stimulate the immune system, such as the widely available immune checkpoint inhibitors. While the choice of cancer treatment can profoundly impact the rates of seroconversion, currently, there is no evidence that patients receiving these agents are at higher risk of adverse events following the administration of COVID-19 vaccination [14].

While patients with haematological malignancies represent a highly susceptible group with an urgent requirement for effective and available vaccines, with the limited data available at this time, it is critical that these individuals continue to maintain ongoing COVID-19 protective measures after vaccination or infection, including wearing face-masks, social distancing and the screening and vaccination of family members. Testing for seroconversion after vaccination or infection may also be warranted.

## References

[1] Richards M, Anderson M, Carter P, Ebert BL, Mossialos E (2020). The impact of the COVID-19 pandemic on cancer care. Nat Cancer..

[2] Thakkar A, Gonzalez-Lugo JD, Goradia N, Gali R, Shapiro LC, Pradhan K (2021). Seroconversion rates following COVID-19 vaccination among patients with cancer. Cancer Cell.

[3] Mehta V, Goel S, Kabarriti R, Cole D, Goldfinger M, Acuna-Villaorduna A (2020). Case fatality rate of cancer patients with COVID-19 in a New York Hospital System. Cancer Discov.

[4] Liang W, Guan W, Chen R, Wang W, Li J, Xu K (2020). Cancer patients in SARS-CoV-2 infection: a nationwide analysis in China. Lancet Oncol.

[5] Robilotti EV, Babady NE, Mead PA, Rolling T, Perez-Johnston R, Bernardes M (2020). Determinants of COVID-19 disease severity in patients with cancer. Nat Med.

[6] Vijenthira A, Gong IY, Fox TA, Booth S, Cook G, Fattizzo B (2020). Outcomes of patients with hematologic malignancies and COVID-19: a systematic review and meta-analysis of 3377 patients. Blood..

[7] Ollila TA, Lu S, Masel R, Zayac A, Paiva K, Rogers RD, et al. Antibody response to COVID-19 vaccination in adults with hematologic malignant disease. JAMA Oncol. 2021;7:1714–6. 10.1001/jamaoncol.2021.4381 PMC8358793 34379085

[8] Cattaneo C, Cancelli V, Imberti L, Dobbs K, Sottini A, Pagani C (2021). Production and persistence of specific antibodies in COVID-19 patients with hematologic malignancies: role of rituximab. Blood Cancer J.

[9] Krammer F (2021). A correlate of protection for SARS-CoV-2 vaccines is urgently needed. Nat Med.

[10] Re D, Seitz-Polski B, Carles M, Brglez V, Graça D, Benzaken S, et al. Humoral and cellular responses after a third dose of BNT162b2 vaccine in patients treated for lymphoid malignancies. medRxiv:2021:2021.07.18.21260669 [Preprint]. 2021 [cited 2021 Jul 22]: [22 p.]. Available from 10.1101/2021.07.18.21260669.

[11] Shaw RH, Stuart A, Greenland M, Liu X, Nguyen Van-Tam JS, Snape MD (2021). Heterologous prime-boost COVID-19 vaccination: initial reactogenicity data. Lancet.

[12] Friese CR, Choueiri TK, Duma N, Farmakiotis D, Grivas P, Rini BI (2021). Care without a compass: Including patients with cancer in COVID-19 studies. Cancer Cell.

[13] Desai A, Gainor JF, Hegde A, Schram AM, Curigliano G, Pal S (2021). COVID-19 vaccine guidance for patients with cancer participating in oncology clinical trials. Nat Rev Clin Oncol.

[14] Waissengrin B, Agbarya A, Safadi E, Padova H, Wolf I (2021). Short-term safety of the BNT162b2 mRNA COVID-19 vaccine in patients with cancer treated with immune checkpoint inhibitors. Lancet Oncol.

